# AMH has no role in predicting oocyte quality in women with advanced age undergoing IVF/ICSI cycles

**DOI:** 10.1038/s41598-020-76543-y

**Published:** 2020-11-12

**Authors:** Xiuliang Dai, Yufeng Wang, Haiyan Yang, Tingting Gao, Chunmei Yu, Fang Cao, Xiyang Xia, Jun Wu, Xianju Zhou, Li Chen

**Affiliations:** 1 Department of Reproductive Medicine Center, The Affiliated Changzhou Maternal and Child Health Care Hospital of Nanjing Medical University, Changzhou, 213000 Jiangsu China; 2 Research Center for Bone and Stem Cells, Department of Anatomy, Histology and Embryology, Nanjing Medical University, Nanjing, 210029 China; 3 Department of Neurology, Integrated Hospital of Traditional Chinese Medicine, Southern Medical University, Guangzhou, 510315 Guangdong China; 4 Department of Neurology, The Affiliated Changzhou No. 2 People’s Hospital of Nanjing Medical University, Changzhou, 213000 Jiangsu China

**Keywords:** Endocrine reproductive disorders, Pregnancy outcome, Infertility

## Abstract

It has been widely acknowledged that anti-Müllerian hormone (AMH) is a golden marker of ovarian reserve. Declined ovarian reserve (DOR), based on experience from reproductive-aged women, refers to both the quantitative and qualitative reduction in oocytes. This view is challenged by a recent study clearly showing that the quality of oocytes is similar in young women undergoing IVF cycles irrespective of the level of AMH. However, it remains elusive whether AMH indicates oocyte quality in women with advanced age (WAA). The aim of this study was to investigate this issue. In the present study, we retrospectively analysed the data generated from a total of 492 IVF/ICSI cycles (from January 2017 to July 2020), and these IVF/ICSI cycles contributed 292 embryo transfer (ET) cycles (from June 2017 to September 2019, data of day 3 ET were included for analysis) in our reproductive centre. Based on the level of AMH, all patients (= > 37 years old) were divided into 2 groups: the AMH high (H) group and the AMH low (L) group. The parameters of in vitro embryo development and clinical outcomes were compared between the two groups. The results showed that women in the L group experienced severe DOR, as demonstrated by a higher rate of primary diagnosis of DOR, lower antral follicle count (AFC), higher level of basal follicle stimulating hormone (FSH) and cancelation cycles, lower level of E2 production on the day of surge, and fewer oocytes and MII oocytes retrieved. Compared with women in the H group, women in the L group showed slightly reduced top embryo formation rate but a similar normal fertilization rate and blastocyst formation rate. More importantly, we found that the rates of implantation, spontaneous miscarriage and livebirth were similar between the two groups, while the pregnancy rate was significantly reduced in the L group compared with the H group. Further analysis indicated that the higher pregnancy rate of women in the H group may be due to more top embryos transferred per cycle. Due to an extremely low implantation potential for transfer of non-top embryos from WAA (= > 37 years old) in our reproductive centre, we assumed that all the embryos that implanted may result from the transfer of top embryos. Based on this observation, we found that the ratio of embryos that successfully implanted or eventually led to a livebirth to top embryos transferred was similar between the H and the L groups. Furthermore, women with clinical pregnancy or livebirth in the H or L group did not show a higher level of serum AMH but were younger than women with non-pregnancy or non-livebirth. Taken together, this study showed that AMH had a limited role in predicting in vitro embryo developmental potential and had no role in predicting the in vivo embryo developmental potential, suggesting that in WAA, AMH should not be used as a marker of oocyte quality. This study supports the view that the accumulation of top embryos via multiple oocyte retrieval times is a good strategy for the treatment of WAA.

## Introduction

Anti-Müllerian hormone (AMH) secreted by granulosa from small growing follicles in ovary, plays a very important role in maintaining the “follicle pool”^1^. Female mice with AMH deficiency exhibit a phenotype of premature depletion of primordial follicles^2^. The mechanism of the action of AMH on maintaining the “follicle pool” is associated with its suppression of genes (encoding stimulatory growth factors) required for the cyclic recruitment of primordial follicles and a decrease in the sensitivity of primordial follicles in response to the stimulation of follicle-stimulating hormone (FSH)^3,4^. Therefore, the level of serum AMH can reflect ovarian reserve.

Currently, AMH has been widely used as a golden maker for evaluating ovarian reserve of females, particularly in the field of assisted reproduction^5,6^. This is because of the high sensitivity of the AMH concentration in reflecting ovarian reserve, which exhibits stable expression that is independent of the menstrual cycle and can be accurately and easily determined in serum^7,8^. Not restricting the ovarian reserve, AMH can also serve as a useful marker in predicting the ovarian response to controlled ovarian stimulation, cycle cancellation and time of menopause^9,10^. However, reports regarding the predictive value of AMH on clinical pregnancy and live birth in assist reproduction are controversial^11–14^. This may be due to inefficient exclusion of confounding factors. For example, when comparing the difference in pregnancy or live birth rate, the quality, quantity and in vitro culture time of embryos transferred (ET) should be considered. Furthermore, the age of females, which has been demonstrated as an independent factor affecting the success of ART, is also an important factor that should be considered^15–17^. Successful pregnancy or live birth in assisted reproduction is determined not only by the quantity but also by the quality of oocytes retrieved. Sometimes, the quality of oocytes is more important. One embryo that can lead to a livebirth is enough for the patient to have a baby. Therefore, we believe that clarifying whether AMH can be treated as a marker reflecting oocyte quality is more important. This will help us to understand the nature of AMH and develop therapeutic strategies scientifically and rationally.

A previous study analysing women (aged 30 to 44 years) without a history of infertility who had been trying to conceive for 3 months or less in a natural way revealed that AMH concentration was not associated with reduced fertility, indicating that AMH concentration is independent of oocyte quality^18^. Consistently, a recent study demonstrated that AMH did not reflect oocyte quality in young patients who underwent IVF cycles^19^. However, it is unclear whether AMH can reflect oocyte quality in women of advanced age (WAA). Therefore, the aim of this study was to investigate this issue.

In this study, we retrospectively analysed the data collected from couples with women aged more than 36 years who underwent the IVF/ICSI/ET cycles in the reproductive centre of Changzhou Maternal and Health Care Hospital. Based on the level of serum AMH, the WAAs were divided into two groups: AMH high (= > 1.1 ng/ml, H) and AMH low (< 1.1 ng/ml, L) groups. The main measurements, including oocytes retrieved, average MII oocytes, the ratio of MII oocytes to total oocytes retrieved, the rate of normal fertilization, top day 3 embryo formation (derived from 2PN-zygotes), pregnancy, implantation, spontaneous miscarriage and live birth, the ratio of embryos that successfully implanted (IPEs) or finally led a livebirth (LPEs) to top embryos transferred, duration of pregnancy and livebirth weight, were compared between the two groups. In addition, the AMH levels between pregnancy and non-pregnancy, and between live birth and non-livebirth in the H and L groups were compared.

## Materials and methods

### Study design

This study was a retrospective study. The patients included in this study who received IVF/ICSI/ET treatment in the reproductive centre of Changzhou Maternal and Health Care Hospital contributed a total of 492 IVF/ICSI cycles (from January 2017 to July 2020) and these IVF cycles-contributed 292 ET cycles (from June 2017 to September 2019). According to the serum level of AMH, the patients were divided into two groups: the H group (= > 1.1 ng/ml) and the L (< 1.1 ng/ml) group. Cancelation rate, baseline characteristics of the cycles and patients, parameters related to in vitro embryo development, characteristics of embryo transfer and clinical outcomes were compared between the H and L groups. In addition, age of the female and the level of AMH in pregnancy and non-pregnancy, or livebirth and non-livebirth in the H or L group were compared, respectively. The number of missing values of parameters in the present study was less than 5; for data analysis, these missing values were ignored.

All of the included patients read and signed the informed consent form. This retrospective study was approved by the Ethics Committee of Changzhou Maternal and Child Health Care Hospital and Nanjing Medical University. All the treatments in the present study were performed strictly in accordance with the Declaration of Helsinki for Medical Research.

### Inclusion criteria

(a) Females aged more than 36 years without a diagnosis of recurrent spontaneous abortion (RSA). It has been reported that females at the age of 35 will commonly experience a sharp decline in fertiltiy^20^. Therefore, infertile females more than 36 years old can be considered WAA. Due to the complex causes of RSA, including females with RSA will affect the accuracy of the analysis of ET-related clinical outcome parameters^21^. Furthermore, it has been implicated that low AMH levels may be associated with an increased risk of embryonic aneuploidy in WAA with RSA^22^.

(b) Exclusion of the couples where either the male or female has chromosomal abnormalities and a history of cancer. Females or males with chromosomal abnormalities or a history of receiving cancer treatment may potentially affect the embryo developmental potential. Furthermore, factors that could affect oocyte quality, including females with habits of smoking and drinking, or females with dyslipidaemia, pelvic infections and diabetes mellitus, were excluded.

(c) Considering the possible impact of the stimulation protocol on oocyte quality, natural cycles were excluded from the analysis.

(d) Only data from IVF/ICSI cycles were included for analysis. Late-ICSI or TESA/PESA ICSI will affect the embryo developmental potential.

(e) ET cycles with a transfer of day 3 embryos were included for analysis. Due to limited oocytes obtained from WAA in the L group, rare cases had extra embryos for further blastocyst culture and subsequent blastocyst transfer.

### Stimulation protocols

#### Long protocol

On day 5 of menstruation, females received desogestrel (0.15 mg) and ethinylestradiol (30 ug) tablets (Marvelon, Organon Pharmaceutical Co Ltd, the Netherlands; one tablet per day for 17 consecutive days). Then, triptorelin acetate (Decapeptyl, Ferring Pharmaceuticals) was used at a dose of 0.1 mg/day until pituitary downregulation was confirmed (no ovarian cysts > 8 mm; E2 < 50 pg/ml). Gonadotropin (Gn, Anhui Fengyuan Pharmaceutical Co., China) was used at an initiative dose ranging from 150–225 U. The late dose of Gn (Anhui Fengyuan Pharmaceutical Co., China) was adjusted according to the size, counts of follicles and hormone levels. When a follicle diameter =  > 18 mm, or two follicles =  > 17 mm, or 3 follicles =  > 16 mm, human chorionic gonadotrophin (HCG, 5000–10,000 IU, Lizhu Pharmaceutical Trading Co., German) was administered for trigger ovulation.

#### Antagonist protocol

On days 2–3 of menstruation, Gn (Anhui Fengyuan Pharmaceutical Co., China) was given at an initiative dose ranging from 150 to 225 U. The late dose of Gn (Anhui Fengyuan Pharmaceutical Co., China) was adjusted according to the size and counts of the follicles and hormone levels. When the diameter of dominant follicles =  > 12–14 mm, GnRH antagonist (0.25 mg/d, Cetrotide, 0.25 mg, Merck Serono, Germany) was administered until the maturity of follicles as mentioned above, and then HCG was used to trigger ovulation.

#### PPOS protocol

PPOS protocol was performed as we described previously^23^. Medroxyprogesterone acetate (MPA; 6 mg/day; Guang Zhou Xianling Pharmaceutical Co., China) and Gn (150–225 IU; Anhui Fengyuan Pharmaceutical Co., China) were administered from day 3 of menstruation onward until the dominant follicle diameter reached > 18 mm. Then, triptorelin (0.1 mg; Decapeptyl, Ferring Pharmaceuticals) and hCG (4000 IU; Lizhu Pharmaceutical Trading Co., German) were administered to trigger ovulation.

#### Mini-stimulation protocol

MPA in the PPOS protocol was substituted by clomiphene (50 mg/day; Cyprus Goth Pharmaceutical Co., Ltd) in the mini-stimulation protocol. The remainder of the procedure was identical to PPOS.

Thirty-six hours later, oocyte retrieval was performed after the trigger.

### Embryo culture procedures

After oocytes retrieval, insemination was performed by the conventional IVF or ICSI method in IVF-plus medium (Vitrolife, Sweden). The observation of pronuclei, embryo culture and score were performed as we described previously^24^. Briefly, 18 h after insemination, zygotic embryos (day 1) with 2 pronuclei were considered normal fertilization and were placed into G1 plus medium (Vitrolife, Sweden) for further culture. On day 3, the morphology of the embryos was evaluated and scored according to a previously described method^24^. Grade I and II embryos were taken as top embryos, while Grade III embryos were taken as non-top embryos. These embryos were frozen by vitrification, transferred into the uterine cavity, or further cultured to the blastocyst stage. For further culture, day 3 embryos were placed into G2-plus medium (Vitrolife, Sweden), and on day 5 or 6, the embryos were morphologically evaluated and scored. The usable blastocysts were frozen by vitrification or transferred into the uterine cavity. Morphological evaluation of embryos in our reproductive centre was performed by Dr Yufeng Wang, who has more than ten years of work experience as an embryologist.

### Statistical analysis

All analyses were performed using GraphPad Prism software (version 8.0.1). The chi-square test was used to compare the data of the constituent ratio. Continuous data were first examined by the Normality and Lognormality test. Data without normally distributed, data were compared by the Mann–Whitney U test. P < 0.05 was considered statistically significant.

## Results

### Numbers of individuals at each stage of the present study

**Figure 1:**
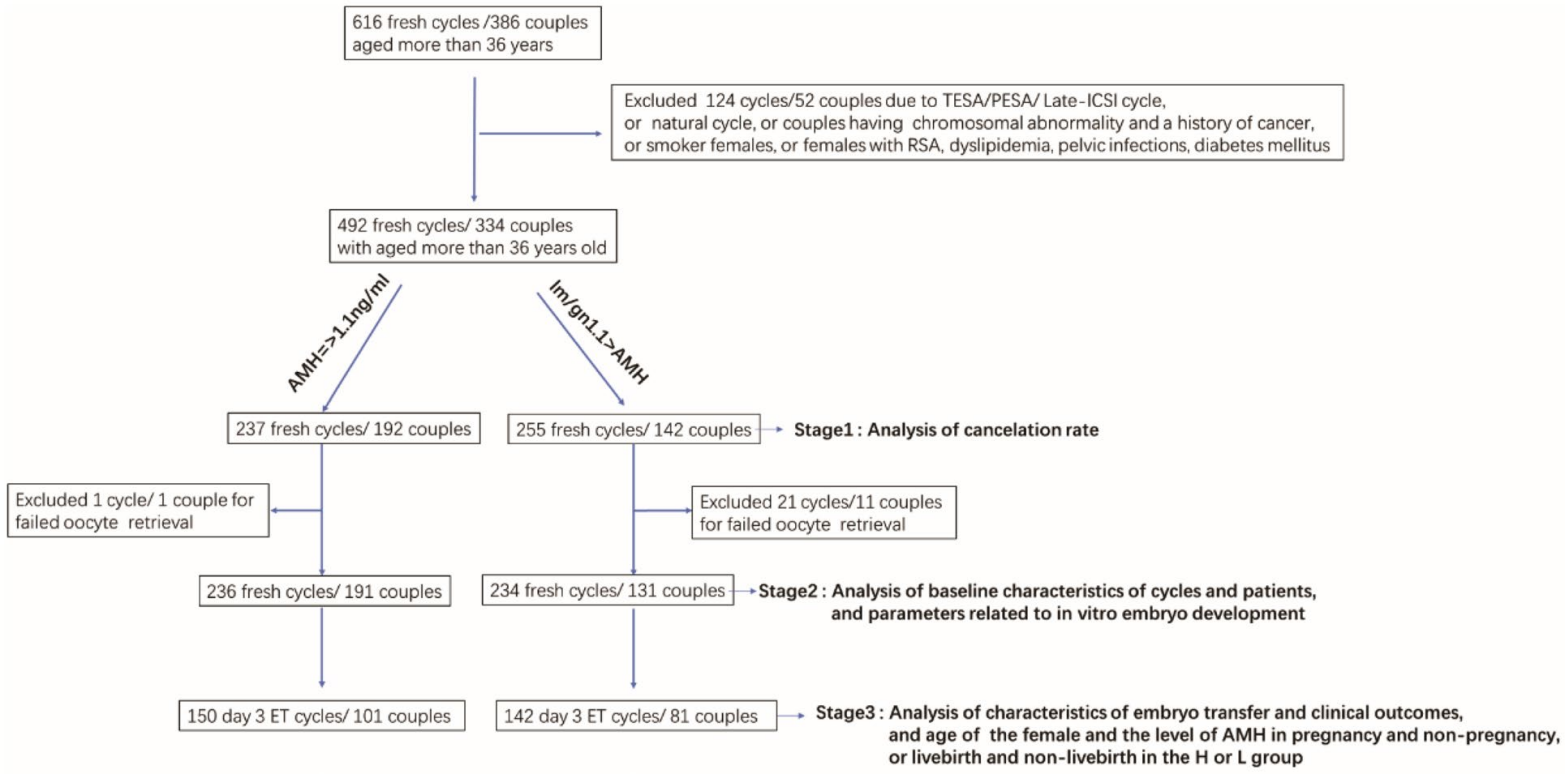
Flow chart of the study design. According to the inclusion criteria, a total of 492 fresh cycles (334 couples) were included for analysis of the cancelation rate. Then, cycles with failed oocyte retrieval were further excluded from analysis of baseline characteristics of cycles and patients, and parameters related to in vitro embryo development. Finally, these fresh cycles contributing day 3 ET cycles were included in order to analyse characteristics of the embryo transfer and clinical outcomes, as well as the age of the female and the level of AMH in pregnancy and non-pregnancy or livebirth and non-livebirth in the H or L group.

The flow chart of the study was described in Fig. 1. Six hundred and sixteen fresh cycles among women aged more than 36 years old (386 couples) were included in the present study. A total of 124 fresh cycles (52 couples) with TESA/PESA/ Late-ICSI cycles, natural cycles, couples with RSA, couples with chromosomal abnormalities or a history of cancer were excluded from the analysis of cancelation rate (Fig. 1). Consequently, a total of 237 fresh cycles (192 couples) in the H group while a total of 255 fresh cycles (142 couples) in the L group were included for the analysis of cancelation rate (Fig. 1). Then, a total of 22 fresh cycles (12 couples) with failed oocyte retrieval were further excluded for analysis of baseline characteristics of the cycles and patients and parameters related to in vitro embryo development (Fig. 1). Consequently, a total of 236 fresh cycles (191couples) in the H group while a total of 234 fresh cycles (131 couples) were included for analysis of baseline characteristics of the cycles and patients and parameters related to in vitro embryo development (Fig. 1). These IVF/ICSI cycles contributing day 3 ET cycles were included in order to analyse characteristics of the embryo transfer and clinical outcomes, as well as the age of the female and the level of AMH in pregnancy and non-pregnancy or livebirth and non-livebirth in the H or L group. A total of 150 day 3 ET cycles (101 couples) were included in the H group while a total of 142 day 3 ET cycles (81 couples) were included in the L group (Fig. 1).

### Baseline characteristics of the cycles and patients

A total of 237 IVF/ICSI cycles were included for analysis in the H group, while 255 cycles were included in the L group (Table 1). The cancelation rate was significantly higher in the L group than in the H group (Table 1). After exclusion for cycles with failed oocyte retrieval, a total of 236 cycles (191 couples) were included in the H group, while 234 cycles (131 couples) were included in the L group (Table 1). Data were collected from these cycles for further analysis. The percentage of patients with single oocyte retrieval was significantly higher, while the percentage of patients with repeated oocyte retrievals was significantly lower in the H group than in the L group (Table 1). The percentage of IVF cycles, age or BMI of males and females, sperm DNA fragmentation index (DFI), percentage of primary infertility and primary diagnosis of other factors, and duration of infertility were comparable between the two groups (Table 1). The percentage of primary diagnoses of DOR, the percentage of ovarian stimulation with the long protocol and antagonist protocol were significantly higher, while AMH levels, AFC, primary diagnosis of tubal factor, percentage of ovarian stimulation with progestin-primed ovarian stimulation (PPOS) and mini-stimulation protocols, total gonadotropin (Gn) dose, basal FSH and E2 level on the surge day were significantly lower in the H group than in the L group (Table 1).

**Table 1:** Baseline characteristics of the cycles and patients.

	H	L	p
Total cycles (n)	237	255	
Cancelation cycles (n)	1	23^#^	
Cancelation rate (%)	1/237 (0.42)	23/255 (9.02)	< 0.0001^a^
Cycles with available oocytes (n)	236	234	
Couples (n)	191	131	
**Couples having single or multiple cycle(s) of oocyte retrieval**			
Single	158/191 (82.72)	66/131 (50.38)	< 0.0001^a^
Multiple (= > 2 cycles)	33/191(17.28)	65/131 (49.62)	< 0.0001^a^
IVF cycles (%)	191/236 (80.93)	200/234 (85.47)	0.1884^a^
AMH (ng/ml)	2.31 [1.54, 3.61]	0.66 [0.46, 0.83]	< 0.0001^b^
AFC (n)	7 [5, 8]	4 [2, 5]	< 0.0001^b^
**Age (years)**			
Female	39 [38, 41]	39.0 [38, 41]	0.5220^b^
Male	40.00 [37, 44]	40.00 [37, 43]	0.3972^b^
**BMI**			
Female	23.6 [21.3, 25.2]	23.1 [21.4, 24.4]	0.1521^b^
Male	24.60 [22.8, 27.35]	25.4[23.1, 27.3]	0.8669^b^
DFI	13.60 [9.21, 21.05]	15.75 [9.67, 22.03]	0.2593^b^
Primary infertility (%)	60/236 (25.42)	59/234 (25.21)	0.9582^a^
Infertility duration (years)	3.00 [1, 8]	3.00 [1, 6]	0.3284^b^
**Primary diagnosis (%)**			
Tubal factor	140/236 (59.32)	76/234 (32.48)	< 0.0001^a^
DOR	22/236 (9.32)	96/234 (41.02)	< 0.0001^a^
Others	74/236 (31.36)	62/234 (26.51)	0.2453^a^
**Simulation protocol**			
Long protocol (%)	105/236 (44.49)	4/234 (0.02)	< 0.0001^a^
Antagonist protocol (%)	56/236 (23.73)	11/234 (0.05)	< 0.0001^a^
PPOS protocol (%)	65/236 (27.54)	140/234 (59.83)	< 0.0001^a^
Mini-stimulation protocol (%)	10/236 (4.24)	79/234 (33.76)	< 0.0001^a^
Basal FSH (IU/L)	6.91[5.77, 7.87]	8.18 [6.74, 10.64]	< 0.0001^b^
Total dose of Gn	2325 [1775, 3000]	2138 [ 1125, 2869]	0.0121^b^
E2 on the day of the surge (ng/L)	2002 [1309, 3220]	1014 [561.2, 1463]	< 0.0001^b^

Data are presented as the median [the first quartile, the third quartile] or count (percentage). # Two cycles were cancelled due to failed useful embryo formation, and the remaining had no available oocytes.

*H* AMH high group; *L* AMH low group; *DFI* DNA fragmentation index; *Gn* gonadotropin; *AFC* antral follicle count; *PPOS* progestin-primed ovarian stimulation; *DOR* declined ovarian reserve.

^a^Chi-squared test.

^b^Mann–Whitney U test.

### Comparison of in vitro embryo developmental parameters between the H and L groups

As shown in Table 2, the average total oocyte retrieval and MII oocytes were reduced significantly, while the rate of MII oocytes to total oocytes retrieved was significantly higher in the L group than in the H group (Table 2). The normal fertilization rate and useful blastocyst formation rate were comparable between the two groups (Table 2). In addition, the rate of top Day 3 embryo formation (derived from 2PN zygotes) was slightly lower in the L group than in the H group (Table 2).

**Table 2:** In vitro embryo development.

	H	L	p
Average oocytes retrieved	7 [5, 10]	3 [2, 4]	< 0.0001^b^
Average MII oocytes	6 [4, 9]	2 [1,4]	< 0.0001^b^
Rate of MII (%)	1658/1809 (91.65)	677/719 (94.16)	0.0323 ^a^
Normal fertilization (%)	1323/1658 (79.8)	540/677 (79.8)	0.9864 ^a^
Top D3 embryos from 2PN (%)	942/1323 (71.2)	356/540 (65.93)	0.0246 ^a^
Usable blastocyst formation (%)	353/712 (49.58)	28/55 (50.91)	0.8492 ^a^

Data are presented as the median [the first quartile, the third quartile] or count (percentage).

Normal fertilization was calculated as the ratio of 2PN zygotes to MII oocytes.

^a^Chi-squared test.

^b^Mann–Whitney U test.

### Comparison of clinical outcome-related parameters between the H and L groups

Because most couples in the L group had limited embryos for further blastocyte culture, only day 3 embryo transfer cycles were included for analysis. A total of 150 ET cycles (101 couples) were included in the H group, while 142 ET cycles (81 couples) were included in the L group (Table 3). The percentage of patients with a single ET transfer was significantly higher, while the percentage of patients with repeated ET transfers was significantly lower in the H group than in the L group (Table 3). We found that the number of embryos per transfer in the H group was significantly higher than that in the L group (Table 3). This difference was more significant when referring to top embryos per transfer between the two groups (Table 3). In addition, cycles with only non-top embryo transfer were much more common in L the group than in the H group (Table 3). None of these cycles in the H and L groups resulted in implantation (data not shown). Consequently, the clinical pregnancy rate was significantly lower in the L group than in the H group (Table 3). However, there were no significant differences in the implantation rate, spontaneous miscarriage rate or livebirth rate between the two groups (Table 3). This made us evaluate the clinical outcomes of non-top embryo transfer. We analysed the cycles with only non-top embryo transfer in women (= > 37 years old) in our reproductive centre from January 2017 to July 2019. There were a total of 25 cases (Supplementary Table S1). We found that only one embryo had a successful implantation, but it failed to produce a livebirth (Supplementary Table S1). Considering the extremely limited embryo developmental potential for non-top embryos in WAA, it was reasonable to assume that all the implantation or livebirths resulted from top embryos. We found that the ratio of embryos that successfully implanted (IPEs) or embryos that successfully led to a livebirth (LPEs) to top embryos transferred was similar in the two groups (Table 3). Furthermore, the duration of pregnancy and birthweight were similar between the two groups (Table 3).

**Table 3:** Characteristics of embryo transfer and clinical outcomes.

	H	L	p
ET cycles (n)	150	142	
Couples (n)	101	81	
**Couples having single or multiple ET cycles (%)**			
Single	65/101 (64.36)	40/81 (49.38)	0.0422 ^b^
Multiple (= > 2 cycles)	36/101 (35.64)	41/81 (50.62)	0.0422 ^b^
Embryos/transfer	2 [2, 2]	2 [1, 2]^a^	0.0019^a^
Top embryos/transfer	2 [1, 2]	1 [1, 2]^a^	< 0.0001^a^
Only grade 3 embryo transfer cycles (n)	5	11	
Clinical pregnancy rate (%)	54/150 (36.00)	35/142 (24.65)	0.0352^b^
Implantation rate (%)	62/276 (22.46)	38/234 (16.24)	0.0777^b^
Spontaneous miscarriage rate (%)	20/54 (37.04)	11/35 (31.43)	0.5875^b^
Live birth rate (%)	34/150 (22.67)	24/142 (19.9)	0.2171^b^
IPEs/ top embryos (%)	62/248 (25.0)	38/191 (18.8)	0.2061^b^
LPEs/ top embryos (%)	38/248 (15.32)	25/191 (13.09)	0.5081^b^
Duration of pregnancy (days)	269.0 [261.3, 275]	269[261.8, 272.8]	> 0.9999^a^
Birthweight (g)	3220 [2578, 3600]	3400[3150, 3650]	0.2139^a^

Data are presented as the median [the first quartile, the third quartile] or count (percentage).

*ET* embryo transfer.

*IPEs* Embryos that were successfully implanted, namely implantation potential embryos.

*LPEs* Embryos that successfully led to a livebirth, namely, livebirth potential embryos.

^a^Mann–Whitney U test.

^b^Chi-squared test.

### Comparison of AMH levels or age of females with pregnancy vs. non-pregnancy or livebirth vs. non-livebirth in the H and L groups

To determine whether females with clinical pregnancy or livebirth showed a higher AMH level than females without clinical pregnancy or livebirth in the H and L groups respectively, we compared the AMH levels in these females. The results showed that no significant difference in AMH levels was observed in pregnancy vs. non-pregnancy or livebirth vs. non-livebirth in either the H or L group (Table 4). Furthermore, we compared the age of females with clinical pregnancy vs. non-clinical pregnancy or livebirth vs. non-livebirth in H and L, respectively. We found that the age of females was significantly younger in the pregnancy or livebirth group than in the non-pregnancy or livebirth group in either the H or L group (Table 4).

**Table 4:** AMH levels or age of females with pregnancy vs. non-pregnancy, or livebirth vs. non-livebirth in the H and L groups, respectively.

	Pregnancy	Non-pregnancy	p	Livebirth	Non-livebirth	p
**H group (n)**	54	96		34	116	
AMH (ng/ml)	2.15 [1.78, 2.77]	2.16 [1.5, 3.76]	0.9271 ^a^	2.13 [1.86, 2.97]	2.16 [1.47, 3.49]	0.5772 ^a^
Age (years)	39 [38, 41]	40 [39, 41]	0.0120 ^a^	38 [37, 39.25]	40 [39, 41]	< 0.0001 ^a^
**L group (n)**	35	107		24	118	
AMH (ng/ml)	0.68 [0.41, 0.87]	0.76 [0.48, 0.93]	0.1656 ^a^	0.62 [0.41, 0.79]	0.76 [0.48, 0.93]	0.1138 ^a^
Age (years)	39 [38,40]	40 [39,42]	0.0031^a^	39 [38,40]	40 [39, 41.25]	0.0120 ^a^

^a^Mann–Whitney U test.

## Discussion

Consistent with a previous report, in the present study, we showed that the level of AMH was strongly correlated with the number of oocytes retrieved and the cycle cancelation rate in WAA^5^. We also found that a low level of AMH in WAA had no impact on the normal fertilization rate but increased the ratio of MII oocytes and decreased the rate of top embryo formation (derived from 2PN zygotes). More importantly, our data suggest that top embryos from WAA with low levels of AMH had the same in vivo developmental potential as top embryos from WAA with relatively high levels of AMH.

Progressive ovarian reserve decline (DOR) with progressive AMH decline is a natural phenomenon during the process of reproductive ageing of females. The traditional view implies that DOR means a reduced quantity and quality of oocytes^25^. However, young females with low AMH also experience DOR. A recent study indicated that the low chance of pregnancy in young females with low levels of AMH undergoing IVF cycles may be due to a reduced quantity but not quality of eggs, raising the question of whether DOR per se represents only the quantitative decline in ovarian reserve^19^. In other words, is it the same thing in WAA? It has been widely accepted that age plays a crucial role in determining the oocyte quality^16^. Therefore, it is unclear whether the low quality of eggs in WAA with low AMH results from age or from low AMH. In the present study, the ages of females in the H group and L group were similar, excluding the influence of female age for further analysis in the present study.

In addition to age, the factors that have been reported to potentially affect the quality of eggs or embryos were similar in the two groups, including BMI of the female or male, age of the male and DFI of semen^26–29^. Furthermore, the percentage of IVF cycles, primary infertility and infertility duration were comparable between the two groups. As expected, compared to the H group, the ovarian reserve was reduced significantly in the L group, as evidenced by a significantly higher rate of primary diagnosis of DOR, lower AFC and higher level of basal FSH. As a consequence, few follicles grew to the mature stage reflected by the low level of E2 on the surge day, resulting in few total or MII oocytes retrieved and a high cancelation rate in the L group. Therefore, more patients with repeated cycles were seen in the L group. It has been reported that no beneficial effects were seen in patients with poor ovarian function receiving a high dose of Gn, and mild stimulation protocols, including PPOS and mini-stimulation protocols, were more appropriate and patient -friendly^30,31^. Therefore, there were more cycles with the mild stimulation protocol and less Gn use in the L group. All these results indicated that WAA with a low level of AMH in the present study had a very limited ovarian reserve.

In the present study, we found that patients with low levels of AMH had a higher rate of MII oocytes. This may be associated with the number of follicles that grow in the ovary in WAA. Compared with a large quantity of oocytes, a few oocytes may obtain more sufficient nutrition from the ovary in WAA to support their maturation. Patients with low or high AMH had a similar ratio of normal fertilization, indicating that the level of AMH was not associated with the process of fertilization in WAA. Further analysis showed that women in the L group had a slightly reduced top embryo formation rate compared with that of women in the H group. However, the blastocyst formation rate in the two groups was similar. Although the difference in the blastocyst formation rate between the H and L groups was not statistically significant, the sample size in the L group was quite small. Therefore, this should be confirmed by future studies including more cases. These results indicated that the level of AMH was not apparently correlated with the embryo in vitro developmental potential in WAA. This finding was consistent with the study showing that young women undergoing IVF cycles have a similar capacity for blastocyst formation irrespective of the level of AMH^19^.

Having a healthy baby is the ultimate goal for ART. Therefore, an embryo that can finally develop into a healthy child is a golden marker reflecting the quality of oocytes^32^. In addition to the livebirth-potential embryo, the implantation-potential embryo and miscarriage-embryo can be used as surrogates of oocyte quality^32^. In the present study, due to more embryos per transfer, particularly more top embryos per transfer in the H group, the women in the H group had a higher pregnancy rate. To our surprise, the implantation rate and spontaneous miscarriage rate together with the most important indicator, namely, the livebirth rate in the two groups were similar. These results urged us to evaluate the underlying cause, namely, the clinical outcomes of non-top embryo transfer in WAA. It has been reported that the clinical pregnancy rate in young women with non-top embryo (day 3) transfer is very low^33^. Therefore, the clinical outcomes in WAA with non-top embryo transfer should be worse. Retrospective analysis of cycles with the transfer of only non-top embryos in women aged more than 36 years in our centre revealed that only one embryo successfully implanted, but failed to lead a livebirth (a total of 25 cycles with non-top embryos transferred). Therefore, we assumed that all the embryos implanted may have resulted from the transfer of top embryos in the present study. Based on this judgement, we calculated the ratio of IPEs or LPEs to total top embryos transferred. We found that the ratio of IPEs or LPEs to top embryos between the two groups was similar. Furthermore, women with clinical pregnancy or livebirth did not show a higher level of serum AMH than women with non-pregnancy or non-livebirth in the H or L group, respectively. These results indicated that the top embryos obtained from WAA had a similar in vivo embryo development potential irrespective of the level of AMH. Although limited cases were included in the present study, we found that the age of females with pregnancies or livebirths was significantly younger than the age of females with non-pregnancy or non-livebirth in either the H or L group. These results were consistent with a previous study demonstrating that age, independent of ovarian reserve, was the main prognostic factor in natural cycle in vitro fertilization^34^, indicating that the results in our study are reliable. Therefore, it should be age rather than AMH that affects the quality of oocytes in WAA.

Taken together, this study showed that oocytes derived from WAA with low levels of AMH had slightly reduced in vitro embryo developmental potential and similar in vivo embryo developmental potential compared to oocytes derived from WAA with relatively high levels of AMH, indicating that DOR per se represents only a decreased quantity but not quality of oocytes in WAA.

As we know, in ART, WAA with diminished ovarian reserve, particularly those with very low levels of AMH, present the most difficult cases for clinicians to address. Clinicians may have a very low expectation of success for these patients, which potentially corrodes the confidence of patients indirectly. The present study revealed that WAA with a very limited ovarian reserve may have the same chance of delivery resulting from every single top embryo (in the present study, approximately 7–8 of top embryos resulted in a livebirth) as WAA with a normal ovarian reserve. Therefore, the therapeutic strategy for these patients should focus on accumulating top embryos via multiple oocyte retrieval events, as a previous study suggested^35^. This should be stated to patients and will thereby help patients consider this situation more scientifically, such as by establishing a suitable level of expectation, reducing anxiety and stress and planning their lives. Therefore, the practical significance of this study is also important.

Several limitations were present in the present study. One is the nature of the respective analysis. Possible confounding factors may not have been taken into consideration in the present study, including the difference in ovarian stimulation protocols and repeated cycles per patient between the two groups. The difference in ovarian stimulation protocols was due to the ineffectiveness of treating patients with limited ovarian reserve by conventional ovarian stimulation protocols with a high dose of Gn, and a mild stimulation protocol is more suitable for these people. In addition, it has been reported that patients with limited ovarian reserve could not benefit from conventional controlled ovarian stimulation as compared to mild stimulation protocol^36^. Therefore, we believe that the difference in the ovarian stimulation protocols between the two groups may not impact the results found in the present study. The reason why we included the repeated cycles was that very few oocytes could be retrieved from WAA with low levels of AMH, and limited cases of WAA with low AMH were available in our reproductive centre. Due to the possible large fluctuation of embryo in vitro developmental parameters from WAA with few oocytes, egg accumulation via multiple ovarian stimulations may help to stabilize our results. Therefore, for these patients, subsequent oocyte retrieval is important as the first time of oocytes retrieval. However, biases from differences in ovarian stimulation protocols and previous failures may exist. Another limitation is that indications drawn in the present study were based on limited cases and data from a single centre. The sample size included in the present study may not be able to discriminate the differences in measurements. Therefore, the conclusions from this study are not definitive but indicative, and these findings need to be confirmed by a large cohort study.

## Supplementary information

Supplementary Information.
