# Supplementary material for: AMH has no role in predicting oocyte quality in women with advanced age undergoing IVF/ICSI cycles
Source: Sci Rep. 2020 Nov 12;10:19750. doi: 10.1038/s41598-020-76543-y (PMC7661530; doi:10.1038/s41598-020-76543-y)
Supplement: Supplementary file 1 — Supplementary Information. [file 41598_2020_76543_MOESM1_ESM.docx]

**AMH has no role in predicting oocyte quality in women with advanced age undergoing IVF/ICSI cycles**

Xiuliang Dai^1,5^, Yufeng Wang^1,5^, Haiyan Yang^1,5^, Tingting Gao^1^, Chunmei Yu^1^, Fang Cao^1^, Xiyang Xia^1^, Jun Wu^2*^, Xianju Zhou^3,4*^, Li Chen^1 *^

^1^Department of Reproductive Medicine Center, The Affiliated Changzhou Maternal and Child Health Care Hospital of Nanjing Medical University, Changzhou, Jiangsu 213000, China

^2^The Research Center for Bone and Stem Cells, Department of Anatomy, Histology and Embryology, Nanjing Medical University, Nanjing, 210029, China

^3^Department of Neurology, Integrated Hospital of Traditional Chinese Medicine, Southern Medical University, Guangzhou, Guangdong510315, China

^4^Department of Neurology, The Affiliated Changzhou No. 2 People’s Hospital of Nanjing Medical University, Changzhou, Jiangsu 213000, China

^5^These authors contributed equally to this work.

^*^Correspondence author:

**Li Chen**,

Department of Reproductive Medicine Center, The Affiliated Changzhou Maternal and Child Health Care Hospital of Nanjing Medical University, Changzhou, Jiangsu, 213000, China.

Email: [CZRCchenli@126.com](mailto:CZRCchenli@126.com)

**Xianju Zhou**,

Department of Neurology, Integrated Hospital of Traditional Chinese Medicine, Southern Medical University, Guangzhou 510315, Guangdong, China or Department of Neurology, The Affiliated Changzhou No. 2 People’s Hospital of Nanjing Medical University, Changzhou, Jiangsu 213000, China.

Email: [xianju_zhou@yahoo.com](mailto:xianju_zhou@yahoo.com)

**Jun Wu,**

The Research Center for Bone and Stem Cells, Department of Anatomy, Histology and Embryology, Nanjing Medical University, Nanjing, 210029, China

Email: wjxue1988@njmu.edu.cn

**Supplementary table I The clinical outcomes of cycles with only non-top embryos transfer in WAA**

| ET cycles (n) | 25 |
| --- | --- |
| Embryos per transfer | 1 [1,2] |
| Pregnancy rate (%) | 1/25 (4.0) |
| Implantation rate (%) | 1/35 (2.9) |
| Miscarriage rate (%) | 1/1 (100) |
| Live birth rate | 0/25 (0) |

Data are presented as the median [the first quartile, the third quartile] or count (percentage).
